# Assessment and Validation of *Globodera pallida* as a Novel In Vivo Model for Studying Alzheimer’s Disease

**DOI:** 10.3390/cells10092481

**Published:** 2021-09-19

**Authors:** Norah A. Althobaiti, Farid Menaa, Aishah E. Albalawi, Johnathan J. Dalzell, Neil D. Warnock, Erin M. Mccammick, Abdulellah Alsolais, Abeer M. Alkhaibari, Brian D. Green

**Affiliations:** 1 Institute for Global Food Security, School of Biological Sciences, Queen’s University Belfast, Belfast BT9 5DL, UK; j.dalzell@qub.ac.uk (J.J.D.); nwarnock03@qub.ac.uk (N.D.W.); emccammick01@qub.ac.uk (E.M.M.); 2 Biology Department, College of Science and Humanities-Al Quwaiiyah, Shaqra University, Al Quwaiiyah 19257, Saudi Arabia; 3 Departments of Internal Medicine and Advanced Technologies, Fluorotronics-California Innovations Corporation, San Diego, CA 92037, USA; 4 Biology Department, Faculty of Science, University of Tabuk, Tabuk 71491, Saudi Arabia; ae.albalawi@ut.edu.sa (A.E.A.); aalkhaibari@ut.edu.sa (A.M.A.); 5 Nursing Department, Faculty of Applied Health Science, Shaqra University, Al Dawadmi 17452, Saudi Arabia; aalsolais@su.edu.sa

**Keywords:** Alzheimer’s disease, amyloid-β, *Globodera pallida*, *Caenorhabditis elegans*, oxidative stress, in vivo model

## Abstract

Background: Whole transgenic or non-transgenic organism model systems allow the screening of pharmacological compounds for protective actions in Alzheimer’s disease (AD). Aim: In this study, a plant parasitic nematode, *Globodera pallida*, which assimilates intact peptides from the external environment, was investigated as a new potential non-transgenic model system of AD. *Methods:* Fresh second-stage juveniles of *G. pallida* were used to measure their chemosensory, perform immunocytochemistry on their neurological structures, evaluate their survival rate, measure reactive oxygen species, and determine total oxidized glutathione to reduced glutathione ratio (GSSG/GSH) levels, before and after treatment with 100 µM of various amyloid beta (Aβ) peptides (1–40, 1–42, 17–42, 17–40, 1–28, or 1–16). Wild-type N2 *C. elegans* (strain N2) was cultured on Nematode Growth Medium and directly used, as control, for chemosensory assays. Results: We demonstrated that: (i) *G. pallida* (unlike *Caenorhabditis elegans*) assimilates amyloid-β (Aβ) peptides which co-localise with its neurological structures; (ii) pre-treatment with various Aβ isoforms (1–40, 1–42, 17–42, 17–40, 1–28, or 1–16) impairs *G. pallida*’s chemotaxis to differing extents; (iii) Aβ peptides reduced survival, increased the production of ROS, and increased GSSG/GSH levels in this model; (iv) this unique model can distinguish differences between different treatment concentrations, durations, and modalities, displaying good sensitivity; (v) clinically approved neuroprotective agents were effective in protecting *G. pallida* from Aβ (1–42) exposure. Taken together, the data indicate that *G. pallida* is an interesting in vivo model with strong potential for discovery of novel bioactive compounds with anti-AD activity.

## Highlights

- *G. pallida*, a plant parasitic nematode, can be used as a non-transgenic model of AD.
- *G. pallida* appears to be a reliable non-transgenic nematode compared to *C. elegans* transgenic strains, for studying AD experimentally.
- *G. pallida* can assimilate amyloid beta (Aβ) peptides, which co-localize with its neurological structures mimicking AD physiopathology.
- Treatment with various Aβ isoforms 1–40, 1–42, 17–42, 17–40, 1–28, or 1–16) impaired *G. pallida*’s chemotaxis, survival, production of ROS, and GSSG/GSH levels.
- *G. pallida* represents a unique model that can sensitively distinguish differences between different treatment concentrations, durations, and other modalities.
- Clinically approved neuroprotective agents were effective in protecting *G. pallida* from Aβ (1–42) exposure.
- *G. pallida* is an interesting new in vivo model with strong potential for discovery of novel bioactive compounds with anti-AD activity.

## 1. Introduction

Alzheimer’s disease (AD) is a progressive neurodegenerative disorder and the leading cause of age-related dementia affecting an estimated 50 million people worldwide [1,2]. The Alzheimer’s Association (2020) have reported that 60 to 80% of dementia patients have AD [3]. Thus, AD represents a global public health concern and has the potential to cause serious economic damage to major economies. The amyloid cascade hypothesis attributes the development of AD pathology to the production and deposition of amyloid-β (Aβ) peptides in the brain [4,5].

Various Aβ isoforms of differing length exist, the longest of which, Aβ (1–42), is the most toxic [6,7]. Animal models of AD which develop Aβ pathology are important in the preclinical testing of new AD therapies but remain impractical and expensive when intended for large-scale drug screening. One alternative for facilitating high-throughput screening (HTS) is to employ cell culture techniques using neuronal primary cells/immortalized cell lines or stem-cells which have been differentiated into neurons [8]. However, the downside of this approach is that cells do not recapitulate the complexity of pathological processes on the level of an organism. Therefore, a more complex alternative is the use of invertebrate transgenic organisms as disease models. Both the fly *Drosophila melanogaster* and the nematode *Caenorhabditis elegans* have been used extensively in studying AD pathology, including the effects of Aβ [9]. The extremely short life cycles of these organisms have allowed scientists to easily perform high-throughput screens for pharmacological compounds in a relatively short timescale and at low cost. Transgenic *C. elegans* has been particularly used to study genetic and biological mechanisms of AD, among other neurodegenerative diseases [10,11,12].

Nevertheless, such transgenic models are not completely without problems. Thereby, a common criticism of invertebrate transgenic AD models is that non-transgenic organisms are not appropriate to be used as controls. Indeed, it is difficult to ascertain whether transgenic expression of any aggregating proteins (instead of Aβ) would have had the same behavioral effects compared to that of the corresponding non-transgenic aggregating proteins or would reflect the natural behavior effects [13]. Transgenic *C. elegans* may exhibit transgenesis leading to extrachromosomal arrays containing hundreds of copies of the transforming DNA [14], which subsequently produce exaggerated phenotypes. Besides, some Aβ-expressing *C. elegans* AD models are muscle-expressed systems [15], which make them unsuitable for modelling AD pathology or Aβ neurotoxicity. Although it has been possible to overcome some of the above problems, including by the achievement of the neuronal expression of Aβ in *C. elegans* [16], there is an urgent need to explore novel non-transgenic models which are lesser complex alternatives than transgenic *C. elegans* models of AD.

As a step in this direction, we investigate a novel non-transgenic model, namely *G. pallida*, a plant parasitic nematode. The proposed *G. pallida* model would have potential advantages over *C. elegans*. First, *G. pallida* is capable of assimilating peptides from the external environment, an innate trait lacking in *C. elegans* [17,18]. Indeed, *G. pallida* takes up peptides through the neuronal amphids of its nervous system, enabling the direct observation of neurotoxicity through the measurement of behavioral and biochemical changes [17]. Second, it would be possible to examine the acute dose-response or concentration-dependent effects on the actions of Aβ in *G. pallida*. Third, as Aβ exists in many forms in the human brain, it becomes feasible to isolate the effects of each individual Aβ species and examine their combined effects in *G. pallida*. Fourth, in terms of HTS of drugs, G. *pallida* model would offer greater speed and convenience over *C. elegans.* Fifth, *G. pallida* is extremely sensitive to neuronal RNAi, which is a powerful tool for studying and manipulating the mechanisms that underlie Aβ toxicity [18,19,20].

Herein, we then decided to explore *G. pallida* as a novel in vivo model for studying AD. Specifically, we checked if (i) *G. pallida* can assimilate specifically Aβ peptides, (ii) these peptides co-localise with neurological structures (e.g., the neural ring), and (iii) the behaviour of this nematode is consequently impaired.

## 2. Materials and Methods

### 2.1. Chemicals and Reagents

All Aβ peptides ((1–40), (1–42), (17–42), (17–40), (1–28) and (1–16)) as well as the cocoa peptide 13L (DNYDNSAGKWWVT) were obtained from GL Biochem Ltd. (Shanghai, China).

Mannitol, galantamine, memantine, caffeine, 2,7-dichlorofluorescein diacetate and phosphate-buffered saline (PBS), and all reagents used in chemotaxis assays for *C. elegans* were obtained from Sigma-Aldrich Co Ltd. (Poole, Dorset, UK). Alamar Blue reagent, agar base and agar slurry were purchased from Fisher Scientific Ltd. (Loughborough, UK).

### 2.2. Methods

#### 2.2.1. Chemosensory Studies with *G. pallida*

Potato cyst nematodes (*G. pallida*) were maintained in potato plants at the Agri-Food and Bioscience Institute (AFBI), Belfast, Northern Ireland. The cysts were hatched in fresh potato root diffusate (PRD) at 16 °C in darkness over ten days. Second-stage juveniles (J2s) were first washed in spring water then used directly in assays.

Chemosensory assays were carried out using a modified protocol of a previously published method [21]. Briefly, this involved the preparation of Petri dishes containing 10 mL of 0.25% agar base (prepared with spring water) which provided an environment for nematodes to move within. Mannitol agar plugs (50 mM, used as an attractant) were prepared and then placed into one side of a Petri dish with a control agar plug (using water instead of mannitol) on the opposite side. Two parallel vertical lines (0.5 cm apart) were marked on the Petri dish lid either side of the center point to form a vertical 1 cm ‘dead zone’ (see Appendix A). One hundred *G. pallida* J2 organisms were then suspended in 10 μL of spring water and spotted onto the midpoint of each dish. Only nematodes outside the center ‘dead zone’ were counted. After 2 h at room temperature (RT), the Chemotaxis Index (CI) [22] describing the movement of the worms either towards or away from mannitol was calculated as follows:

(1) $$\mathrm{CI}=\frac{\left(+\mathrm{ve}\right)-\left(-\mathrm{ve}\right)}{\left(+\mathrm{ve}\right)+\left(-\mathrm{ve}\right)}$$

where +ve is the number of organisms on the positive (mannitol) side of the dish, and −ve is the number of organisms on the negative (control) side.

**Figure 1:**
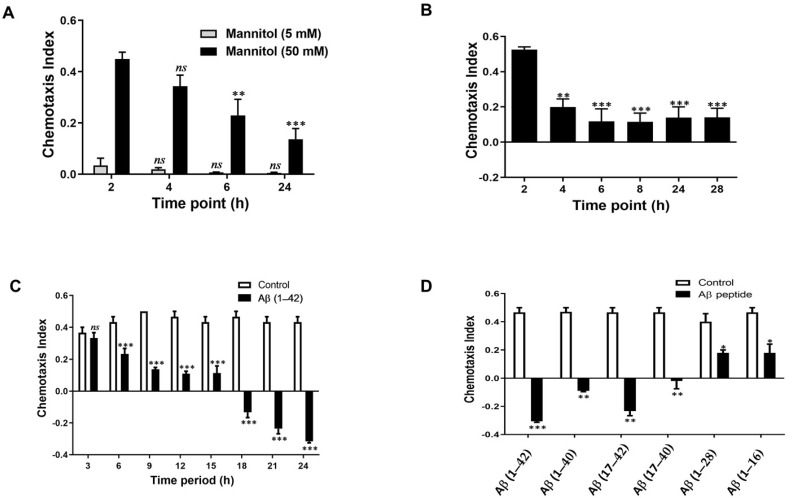
Optimising assays for chemotaxis index (CI) of *G. pallida* in function of (**A**) different concentrations of mannitol (chemoattractant) with CI measured at 2, 4, 6, and 24 h; (**B**) a range of incubation periods in response to mannitol (50 mM). *G. pallida* had the greatest CI to mannitol at 2 h; this effect gradually diminished with increasing incubation time; (**C**) the duration of the Aβ (1–42) pre-incubation, which determines the extent to which the CI was affected. Pre-incubations as short as 6 h significantly affected CI, with the most pronounced effects observed at 18 h and 24 h; (**D**) Aβ fragment peptides in untreated *G. pallida*. The data are mean ± SEM (*n* = 3). *ns* (not significant), * *p* < 0.05, ** *p* < 0.01, and *** *p* < 0.001, compared with control.

**Figure 2:**
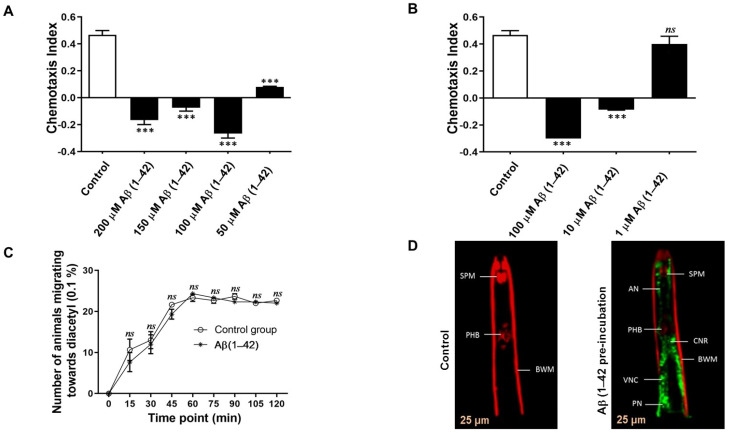
24 h exposure to amyloid-β (Aβ) (1–42) affects *G. pallida* chemosensing in a concentration-dependent manner, but the chemosensing of *C. elegans* is not affected. (**A**) Compared with the vehicle control (1% DMSO in ddH_2_O), Aβ (1–42) concentrations between 50 and 200 µM affected the chemotaxis Index (CI) of *G. pallida* to mannitol; (**B**) It is demonstrated that CI was affected at Aβ (1–42) concentrations as low as 10 µM (but not 1 µM). Approximately 100 *G. pallida* organisms were used per observation (**A**,**B**). (**C**) Groups of *C. elegans* were pre-incubated in the vehicle control or with Aβ (1–42) at concentration of 100 μM, and their migration response to chemoattractant (0.1% diacetyl dissolved in 0.99% ethanol) was measured. *C. elegans* was unaffected. Approximately 30 nematodes (wild-type N2—synchronised young adult age) per observation. Data demonstrated that Aβ (1–42) had no effect on the chemotaxis response of *C. elegans* towards the attractant compared with the vehicle control; (**D**) Confocal microscopy of amyloid-β (Aβ) (1–42)-treated *G. pallida*. *G. pallida* pre-incubated for 24 h with Aβ (1–42) showed Aβ-specific fluorescence staining (green) which was not co-localised with muscle staining (red). In second-stage juveniles (J2s), Aβ (1–42) was in the ‘brain’, i.e., the circumpharyngeal nerve ring (CNR), posterior to the pharyngeal bulb (PHB), and in amphid neurons (AN) running anteriorly towards the stylet protractor muscles (SPM) and parallel to the body wall muscle (BWM). It is also shown in the ventral nerve cord (VNC) and in phasmid neurons (PN). Scale bars = 25 µm. The data are mean ± SEM (*n* = 3). *ns* (not significant), and *** *p* < 0.001, compared with control.

**Figure 3:**
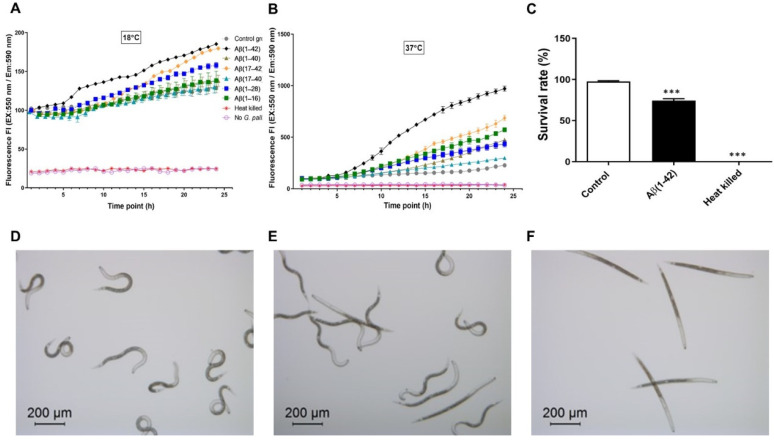
Changes in mitochondrial reductase activity in *G. pallida* in response to amyloid-β (Aβ) peptide fragments. *G. pallida* were incubated with Alamar Blue either at a temperature of (**A**) 18 °C or (**B**) 37 °C, and the relevant Aβ fragment with fluorescence (Ex: 550 nm, Em: 590 nm) was measured every 30 min for 24 h (FLUOstar Omega microplate reader). Aβ (1–42) increased reductase activity by the greatest extent. Representative light microscopy images of *G. pallida* second-stage juveniles (J2s) following 24 h exposure to amyloid-β Aβ (1–42); (**C**) shows percent survival rate using counting by visual observation; (**D**) shows untreated worms (control) with characteristic ‘body bends’; (**E**) shows the Aβ (1–42)-treated group with mixed ‘body bends’ and ‘poker straight’ shape characteristics; (**F**) shows heat-killed worms with characteristic ‘poker straight’ shape. The data are mean ± SEM (*n* = 3) with approximately 100 *G. pallida* organisms per observation. *** *p* < 0.001, compared with control.

**Figure 4:**
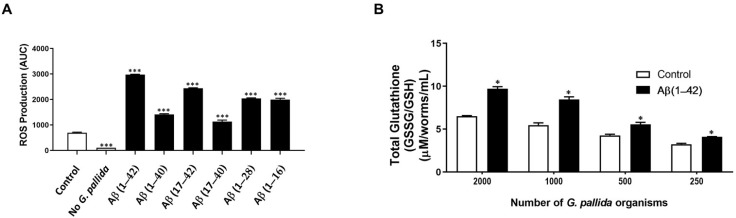
Changes in oxidative stress in *G. pallida* in response to amyloid-β (Aβ) peptide fragments. Total ROS production was determined by adding 2′,7′-dichlorofluorescin diacetate (DCFH-DA) and measuring fluorescence every 30 min for 24 h (Ex: 485 nm, Em: 520 nm, FLUOstar Omega) at RT. (**A**) ROS production for 0–24 h (s) as measured by AUC analysis. Aβ (1–42) increased oxidative stress by the greatest extent; (**B**) Effects of amyloid-β (Aβ) (1–42) on glutathione levels in *G. pallida.* The ratio of GSSG to GSH in cells is a measure of cellular oxidative stress. An OxiSelect™ Total Glutathione (GSSG/GSH) Assay kit (Cell Biolabs, Inc., San Diego, CA, USA) was used for measurements. Treatment of either 250, 500, 1000, or 2000 *G. pallida* second-stage juveniles (J2s) with Aβ (1–42) led to significantly higher GSSG/GSH compared with control. The data for (**A**) are mean ± SEM (*n* = 3) with approximately 100 *G. pallida* organisms per observation; *** *p* < 0.001, compared with control group. The data for (**B**) are mean ± SEM (*n* = 3); * *p* < 0.05, compared with control.

**Figure 5:**
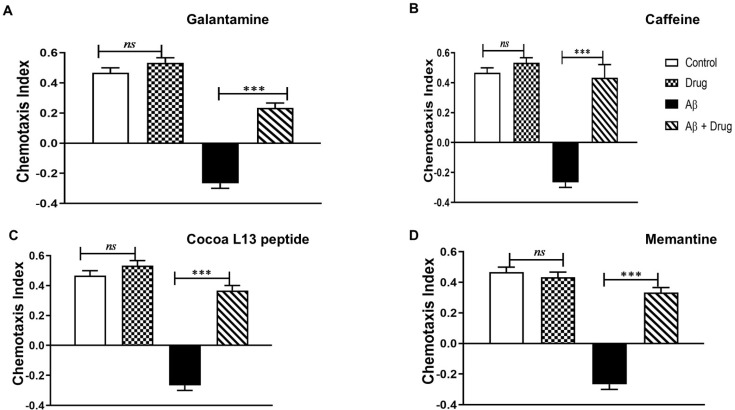
Neuroprotective agents ameliorate Aβ (1–42)-induced impairment of chemosensing. Two clinically used drugs (galantamine and memantine) and two neuroprotective agents (caffeine and 13L cocoa peptide, previously observed to be beneficial in Aβ-expressing *C. elegans*) were selected and assessed for their ability to ameliorate the effects of Aβ (1–42) in *G. pallida*. *G. pallida* second-stage juveniles (J2s) were pre-incubated (24 h) with either (1) the vehicle control (1% DMSO in ddH_2_O), (2) Aβ (1–42), (3) the tested drug alone, or 4) Aβ (1–42) in combination with the tested drug: (**A**) galantamine (100 µM); (**B**) caffeine (3.6 mM); (**C**) 13L cocoa peptide (200 µg/mL); (**D**) memantine (10 mM). When tested alone, none of these agents affected the chemotaxis index (CI). All ameliorated Aβ (1–42)-induced impairments in the CI (*p* < 0.001). The data are mean ± SEM (*n* = 3) with approximately 100 *G. pallida* organisms per observation. *ns* (not significant), *** *p* < 0.001 compared to either the vehicle control or the Aβ (1–42) group.

For Aβ pre-treatments (16 °C; 24 h), approximately 100 *G. pallida* J2 organisms were washed in spring water to remove PRD liquid and then centrifuged (2500 rpm; 2 min). Pellets were reconstituted in 196 µL of spring water and transferred to a 24-well plate; 4 µL of either Aβ (5 mM; 1% dimethyl sulfoxide (DMSO) in double-distilled water (ddH_2_O)) or the vehicle control (1% DMSO in ddH_2_O) was then added to achieve a final peptide concentration of 100 µM of Aβ (1–42), Aβ (1–40), Aβ (17–42), Aβ (17–40), Aβ (1–28) or Aβ (1–16). Studies with Aβ (1–42) were conducted with a range of pre-treatment temperatures (16 °C; 4 °C), periods (3 h to 24 h), and concentrations (1–200 µM). Drug treatments, at the concentrations indicated in Figures 1–5 (i.e., galantamine (100 µM), caffeine (3.6 mM), memantine (10 mM), or 13L cocoa peptide (200 µg/mL), were co-incubated with Aβ (1–42) (100 µM; 16 °C; 24 h).

#### 2.2.2. Immunocytochemistry (ICC)

Approximately 1000 freshly hatched *G. pallida* J2s were immuno-stained using indirect immunofluorescence technique [23]. Organisms were incubated for 24 h either as control or peptide-treated groups, after which they were fixed with 4% paraformaldehyde (PFA) in 0.1 M phosphate-buffered saline (PBS) and incubated in a rotator at 4 °C for 24 h.

Immunocytochemical staining employed purified monoclonal anti-Aβ (1–16) primary antibody 6E10, Isotype: Mouse IgG1 (Biolegend, Inc., San Diego, CA, USA), goat anti-mouse Alexa Fluor 488 (Molecular Probes) pre-adsorbed IgG (H + L) Secondary Antibody, and Alexa Fluor^®^ 488 conjugate (Thermo Fisher Scientific Corp., UK) with fixed worms [24]. Thereby, following a wash in the antibody diluent (AbD; PBS pH 7.4, 0.1% (*v/v*), Triton X-100, 0.1% (*w/v*), sodium azide (NaN_3_) and 0.1% (*w/v*) bovine serum albumin (BSA)), the specimens were incubated at 4 °C in the primary antibody (1:700 dilution in AbD) for three days. Then, a wash in AbD for 24 h preceded the incubation of the specimens with the secondary antibody conjugated to the fluorophore goat anti-mouse Alexa Fluor 488 (Molecular Probes) for 72 h at 4 °C. Subsequently, an additional wash in AbD for 24 h was followed by incubation in tetramethylrhodamine isothiocyanate (TRITC)-conjugated phalloidin for 24 h at 4 °C to visualise endogenous muscle systems (Sigma-Aldrich Co Ltd., Poole, Dorset, UK). After a final wash in AbD for 24 h at 4 °C, the specimens were mounted on glass slides with glycerol/PBS and viewed under the Leica TCS SP5 confocal scanning laser microscope.

#### 2.2.3. Measuring Effects of Aβ on the Health of *G. pallida* Survival Rate

The number of live and dead *G. pallida* J2 organisms was manually counted [25], based on their movements, using a Leica microscope (Leica model: M205C) and the software package Leica Application Suite (LAS). The assays were blind counted by a second party to minimise experimental bias.

#### Measurement of *G. pallida* Viability

Alamar Blue reagent (Life Technologies Ltd., Paisley, Inchinnan, UK) was used to assess the cell viability of *G. pallida* J2 organisms. The Alamar Blue assay is based on the ability of viable cells to produce formazan from the cleavage of tetrazolium salt by functional mitochondria. The Alamar Blue assay was carried out according to manufacturer’s instructions. Briefly, a 96-well plate containing *G. pallida* and the Aβ peptides to be tested was prepared using standard methods, and Alamar Blue was added directly to each well. The plates were then incubated at two different temperatures points, 18 °C (ambient/RT) and 37 °C (physiological), to allow cells to convert resazurin to resorufin, and the signal was measured. Fluorescence was quantified every 30 min for 24 h at Ex550 nm and Em590 nm using a FLUOstar Omega microplate reader, and a mean value was obtained for three independent experiments.

#### Measurement of ROS

The dichlorofluorescein-diacetate (DCF-DA) assay was used to measure the amount of ROS production in *G. pallida* in response to peptides Aβ (1–42), Aβ (1–40), Aβ (17–42), Aβ (17–40), Aβ (1–28), Aβ (1–16), or to the vehicle control. The method used a modified protocol based on previous studies with *C. elegans* [26,27,28]. ROS production was calculated by area-under-the-curve (AUC) analysis.

#### Determination of Total Glutathione

The content of reduced glutathione (GSH) and oxidized glutathione (GSSG) in *G. pallida* was determined using an OxiSelect^TM^ Total Glutathione Assay Kit (Cell BIOLABS, San Diego, CA, USA) in accordance with the manufacturer’s instructions.

#### 2.2.4. *C. elegans* Chemotaxis Assays

Wild-type N2 *C. elegans* (strain N2) was obtained from the Caenorhabditis Genetics 91 Centre, University of Minnesota, Minneapolis, USA, and was cultured on Nematode Growth Medium. Chemosensory assays were performed as described by [29] with some minor modifications.

### 2.3. Data Analysis

All data are expressed as mean ± standard error mean (SEM) (*n* = 3). Data were statistically analysed by a one-way ANOVA with Tukey’s multiple comparison test to compare differences between groups: *ns* (not significant), * *p* < 0.05, ** *p* < 0.01, *** *p* < 0.001. All statistical analyses were performed using Graphpad Prism 5.0 software (GraphPad, San Diego, CA, USA).

## 3. Results

### 3.1. Optimisation of Chemosensory Assays

The optimal concentration of chemoattractant was determined by measuring the chemotaxis index (CI) of *G. pallida* for two different concentrations of mannitol at 2, 4, 6, and 24 h.

As shown in Figure 1A, a 50 mM concentration of mannitol (CI: 0.45 ± 0.05) was highly effective in attracting the nematodes, whereas mannitol at the lower concentration of 5 mM was not (CI: 0.03 ± 0.08).

Next, the optimal time for chemosensory assays was determined. As shown in Figure 1B, the CI value for *G. pallida* was highest at 2 h (CI: 0.52 ± 0.06) and gradually diminished with increasing incubation time.

### 3.2. Effects of Aβ on the Chemotaxis of G. pallida

The effect of Aβ exposure on the chemotaxis of *G. pallida* was evaluated using the optimized concentration mannitol of 50 mM.

Figure 1C shows that the duration of Aβ (1–42) exposure affected the extent to which the CI changed. Indeed, treatments as short as 6 h significantly affected the CI (0.23 ± 0.03; *p* < 0.001), with the most pronounced effects observed at 21 h (−0.23 ± 0.06; *p* < 0.001) and 24 h (−0.31 ± 0.02; *p* < 0.001) compared with the vehicle control group (0.47 ± 0.03).

A pre-incubation time of 24 h was deemed optimal where the response was maximized. Figure 1D shows that pre-incubation (24 h) of *G. pallida* with different Aβ fragment peptides affected its chemoattraction to mannitol (50 mM; 2 h). At a concentration of 100 μM, the CI value was significantly altered for Aβ (1–42), Aβ (1–40), Aβ (17–42), Aβ (17–40), Aβ (1–28), and Aβ (1–16) compared with the vehicle control (CI: 0.47 ± 0.06). The greatest effect on the CI was observed for Aβ (1–42) peptides (CI: −0.30 ± 0.00; *p* < 0.001), followed by Aβ (17–42) (CI: −0.23 ± 0.07), Aβ (1–40) (CI: −0.09 ± 0.04), Aβ (17–40) (CI: −0.02 ± 0.04), Aβ (1–28) (CI: 0.18 ± 0.02) and Aβ (1–16) (CI: 0.18 ± 0.06).

The effects of Aβ (1–42) on the chemotaxis response of *G. pallida* were found to be dependent on the peptide concentration (Figure 2A). Concentrations of Aβ (1–42) from 50 to 200 µM were tested; all induced dysfunction in the CI of *G. pallida* (CI: 200 µM −0.16 ± 0.02, 150 µM −0.07 ± 0.06, 100 µM −0.26 ± 0.08, and 50 µM 0.08 ± 0.05; *p* < 0.001) compared with the vehicle control (CI: 0.52 ± 0.02). Further studies involving 10-fold dilutions of Aβ (1–42) (Figure 2B) demonstrated that 100 µM had the strongest effect on the CI (−0.3 ± 0.06; *p* < 0.001), followed by 10 µM (−0.08 ± 0.05; *p* < 0.001), whereas 1 µM had no significant effect on CI compared with the vehicle control (CI: 0.4 ± 0.05; ns).

Additional studies were carried out to demonstrate that Aβ-induced impairment of chemosensing by *G. pallida* was not evident in *C. elegans*. Chemosensory assays were carried out for *C. elegans* wild-type N2 using 0.1% diacetyl as an attractant. Organisms were exposed to Aβ (1–42) for 24 h. Figure 2C shows that Aβ (1–42) did not cause chemotaxis dysfunction in *C. elegans.* No significant differences between Aβ (1–42)-treated organisms and the vehicle control could be detected at any time point over a 120-min period.

### 3.3. Localisation of Aβ (1–42) within G. pallida

Immunocytochemistry studies were conducted to confirm the capacity of *G. pallida* to assimilate Aβ (1–42), and to establish in which physiological structures of *G. pallida* the peptides are localised.

Figure 2D shows confocal microscographs comparing *G. pallida* exposed to the vehicle control (left) or Aβ (1–42) (right). Aβ (1–42)-treatment (100 µM) produced clear Aβ-specific fluorescence staining (green) which was not co-localised with muscle staining (red). Aβ (1–42) was extensively located throughout the nervous system of *G. pallida* J2s. It was particularly abundant in the amphid and phasmid neurons and in the ‘brain’ i.e., the longitudinal nerve cords of the circumpharyngeal nerve ring (CNR) of *G. pallida* J2s. This confirms the hypothesis that these nematodes can assimilate peptides from the external environment. Furthermore, it is evident that the chemotaxis dysfunctions exhibited in pre-incubation of Aβ (1–42) with *G. pallida* were mediated by the nervous system and not by muscle action. It appears that Aβ (1–42) is localised in both the paired neurons within the central nerve ring and the sensory amphid neurons of *G. pallida* J2s.

### 3.4. Effects of Aβ (1–42) on the Health Parameters of G. pallida

#### 3.4.1. Viability

Figure 3A,B show the effects of Aβ (1–42), Aβ (1–40), Aβ (17–42), Aβ (17–40), Aβ (1–28), and Aβ (1–16) peptide fragments on the viability of *G. pallida*, at 18 °C and 37 °C, respectively, as measured by mitochondrial reductase activity (Alamar Blue). 100 µM of Aβ peptide fragments was used. These effects were compared to the vehicle control.

Aβ (1–42) increased mitochondrial reductase activity by 26.33% at 18 °C and by 255% at 37 °C. Aβ (1–40) caused a negligible change in this activity (1.51%) at 18 °C but increased it drastically by 53% at 37 °C. Aβ (17–42) increased this activity by 12.95% and 120% at 18 °C and 37 °C, respectively. Aβ (17–40) reduced this activity by 4.48% at 18 °C but increased it by 23% at 37 °C. Aβ (1–28) increased this activity by 10.15% and 71% at 18 °C and 37 °C, respectively. Eventually, Aβ (1–16) slightly increased the mitochondrial reductase activity by 6.74% at 18 °C, it is worth noting that this activity increased drastically by 99% at 37 °C.

#### 3.4.2. Survival

100 µM of Aβ peptide fragments was used. Figure 3C indicated the survival of *G. pallida* based on counting using visual observation. Specifically, 24 h exposure of *G. pallida* to Aβ (1–42) significantly decreased the survival rate of *G. pallida* (74.3 ± 0.66%) compared with the vehicle control (97.66 ± 0.88%).

Figure 3D–F represent light microscopy images of *G. pallida* J2s following 24 h exposure to amyloid-β Aβ (1–42). Figure 3D shows untreated worms (used as control) with characteristic ‘body bends’, Figure 3E shows the Aβ (1–42)-treated group with a mix of ‘body bends’ and ‘poker straight’ shape, and Figure 3F shows heat-killed worms with a characteristic ‘poker straight’ shape.

#### 3.4.3. ROS Production

Figure 4A shows that over the 24 h period, all Aβ peptide fragments (100 µM) significantly increased ROS levels in *G. pallida* compared with the vehicle control. Aβ (1–42) increased ROS production to the greatest extent with 329%, followed by Aβ (17–42) with 251%, Aβ (1–28) with 194%, and Aβ (1–16) with 188%. Aβ (1–40) and Aβ (17–40) affected ROS production the least, by 99% and 62%, respectively. When *G. pallida* were not subject to any incubations, there was significantly less ROS production, indicating that these organisms maintain a basal level of ROS.

#### 3.4.4. GSSG/GSH Levels

The ratio of oxidized glutathione (GSSG) to reduced glutathione (GSH) within cells is believed to be a measure of cellular oxidative stress.

Therefore, glutathione levels in *G. pallida* treated with 100 µM Aβ (1–42) for 24 h were measured (Figure 4B). Briefly, the data demonstrated that Aβ (1–42) led to significantly higher GSSG/GSH compared to the control. Moreover, this held true as the number of *G. pallida* J2s varied. The increase was 3.21 µM/worms: J2s = 2000, 2.99 µM/worms: J2s = 1000, 2.32 µM/worms: J2s = 500, and 0.86 µM/worms: J2s= 250 (*p* < 0.001).

### 3.5. Effects of Neuroprotective Agents on Aβ (1–42)-Induced Impairment of Chemosensing

One of the key questions we asked was whether it is possible to prevent Aβ-induced impairments, by testing potential anti-AD drugs exerting specifically anti-Aβ (1–42)-induced impairment of the CI when *G. pallida* is used as a novel in vivo invertebrate model of AD (Figure 5).

Among anti-AD drugs, galantamine is an acetylcholinesterase inhibitor (AChEI) commonly used clinically to treat mild-to-moderate AD [30]. The impact of galantamine in reversing the effects of Aβ (1–42) used at 100 µM on *G. pallida* J2s is shown in Figure 5A. *G. pallida* J2s were pre-incubated (24 h) with either the vehicle control, galantamine (100 μM), Aβ (1–42), or Aβ (1–42) in combination with 100 μM of galantamine. Insignificant difference was noticed between the vehicle control group (CI: 0.50 ± 0.02) and the group treated with just galantamine at 100 μM (CI: 0.53 ± 0.03). However, when galantamine is combined to Aβ (1–42), significant improvements in CI values (CI: 100 μM: 0.31 ± 0.03; *p* < 0.001 and 10 μM: −0.023 ± 0.02; *p* < 0.01) were found compared to that of the Aβ (1–42)-treated group (CI: −0.3 ± 0.01).

We also evaluated other neuroprotective agents, i.e., caffeine (Figure 5B), 13L cocoa peptide (Figure 5C), memantine (Figure 5D), to determine their ability to ameliorate Aβ (1–42)-induced impairment of chemotaxis in *G. pallida* J2s (Figure 5B–D). These neuroprotective agents were selected based on their effectiveness in the transgenic *C. elegans* AD model.

*G. pallida* J2s were pre-incubated (24 h) with either the vehicle control, Aβ (1–42) at 100 µM or Aβ (1–42) at 100 µM in combination with caffeine (3.6 mM), 13L cocoa peptide (200 µg/mL), or memantine (10 mM). When tested alone in *G. pallida*, none of these agents significantly affected the CI (CI: 0.43 ± 0.20 caffeine; CI: 0.51 ± 0.11 13L cocoa peptide; CI: 0.53 ± 0.10 memantine) compared to the vehicle control group (CI: 0.5 ± 0.04). However, all of them did ameliorate Aβ (1–42)-induced impairments in CI (*p* < 0.001). Indeed, all these agents in combination with Aβ (1–42), a significant attenuation in the chemotaxis responses was noticed (CI: 0.33 ± 0.02 caffeine; CI: 0.36 ± 0.04 13L cocoa peptide; CI: 0.43 ± 0.0 memantine) compared to the Aβ (1–42)-treated group (CI: −0.3 ± 0.02; *p* < 0.001).

## 4. Discussion

The principal aim of this investigation was to develop a novel non-transgenic model as a less complicated alternative to *C. elegans* models of AD. For this purpose, the innate ability of the plant parasitic nematode *G. pallida*, to assimilate intact peptides from the external environment into neuronal structures [17,18,31], was exploited.

Aβ-induced toxicity is one of the central mechanisms thought to be involved in the induction of AD pathology [32]. It has been established that synaptic and neuronal degeneration is triggered by Aβ toxicity in the brains of AD patients [33]. Therefore, we investigated the effects of Aβ peptide fragments in *G. pallida*, in anticipation that it could provide new insights into AD mechanisms or assist with the identification of new therapeutic targets. There are several potential advantages to this approach, not least that it allows the direct observation of Aβ toxicity through the measurement of behavioral changes on sensory neurons of this nematode [17,18].

We applied conventional chemosensory assays to objectively assess Aβ-induced effects on the behavior of *G. pallida* J2s. We determined that a 2 h period of chemotaxis towards mannitol as a chemoattractant was optimal. The investigation then turned to how *G. pallida* responded to a range of human Aβ fragments. All Aβ peptide fragments (Aβ (1–42), Aβ (1–40), Aβ (17–42), Aβ (17–40), Aβ (1–28) and Aβ (1–16)) tested in this study significantly affected the chemotaxis index (CI) of *G. pallida*. However, Aβ (1–42) had the most marked effect on CI compared with the vehicle control. In contrast, Aβ (1–28) and Aβ (1–16) exerted the least effect on the CI. These findings corroborate earlier studies showing that Aβ (1–42) is the most toxic in human AD [34,35], and strongly support the proposed causative role of Aβ (1–42)-induced oxidative stress and neurodegeneration in AD [36].

The Aβ peptide is derived from a single transmembrane protein, known as the amyloid precursor protein (APP), and production of Aβ is completed by the sequential actions of two enzymes, β-secretase (BACE) and γ-secretase. The Aβ (1–40) and Aβ (1–42) species are components of the plaques implicated in AD progression. With Aβ (1–42) established as the most potent peptide in our model system, we examined how the duration of Aβ (1–42) treatment affected the CI. We found that incubations of as little as 6 h significantly affected the CI, with the most pronounced effects were observed at 18 h and 24 h. These time-related changes may reflect the typical transit time required for assimilated peptides to reach the nervous system of *G. pallida.* These data confirmed that this nematode can assimilate peptides through chemosensing amphid neurons within cells of the central nerve ring of its nervous system [17,18,21]. In line with our data, it takes typically around 18–24 h for peptides to be absorbed and to impact on the neurons and cellular behaviors of *G. pallida* [17,18,31]. The most likely route for exogenous Aβ assimilation is via retrograde transport along the chemosensory amphid neurons. It is known that other neuropeptides can accumulate within cells of the central nerve ring eliciting strong physiological effects when they interact with receptors on nearby cells [31]. In the case of Aβ we cannot be definitive about whether it is acting intracellularly or extracellularly.

Importantly, we then confirmed that Aβ-induced impairments of chemosensing of *G. pallida* are not recapitulated in *C. elegans*. Wild-type *C. elegans* was not affected by exposure to Aβ (1–42), further demonstrating that *G. pallida’s* known ability to assimilate peptides was critical for sensory impairment to occur. It should be noted that *G. pallida* and *C. elegans* respond to different chemoattractants and therefore a direct like-for-like comparison is not possible.

Next, the sensitivity of *G. pallida*, as an original in vivo non-transgenic model system for AD studies, was assessed by gauging whether Aβ (1–42) affected chemotaxis in a concentration-dependent manner. Concentrations of between 50 and 200 µM of Aβ (1–42) profoundly affected the CI of *G. pallida* compared with the vehicle control. Even Aβ (1–42) concentrations as low as 10 µM had a significant impact on the CI. However, very low Aβ (1–42) concentrations (1 µM) were completely ineffective, showing the relative sensitivity of our method.

We then probed putative mechanisms through which Aβ peptides could be impairing chemotaxis. Mitochondrial dysfunction and oxidative stress contribute to ageing and to the occurrence and progression of AD [37]. Oxidative stress results from ROS, key hallmarks, generated during mitochondrial oxidative metabolism that leads to acceleration of AD pathology [38]. Thus, we evaluated if mitochondrial reductase activity and ROS production could be impacted in *G. pallida* in response to each of the tested Aβ peptide fragments. There is growing evidence that Aβ peptides play a role in increasing ROS production and oxidative stress in AD. Moreover, Aβ induces oxidative damage to the vital cellular biomolecules, DNA, proteins, and lipids oxidation, which are associated with AD pathology [34,39,40,41,42,43]. Many of the tested Aβ peptides significantly increased mitochondrial reductase activity and ROS. Notably, Aβ (1–42) and Aβ (17–42)) most profoundly affected these levels while impacting chemotaxis the most. It is known that human Aβ (1–42) and Aβ (17–42) are toxic [44]. Indeed, Aβ (1–42) is the most toxic of Aβ fragments both in vitro and in vivo, and the methionine amino acid residue located at position 35 of Aβ (1–42) appears to be an important factor. Indeed, this methionine residue would modulate the neurotoxic properties of Aβ (1–42) and the induction of oxidative stress in AD pathology [34,41,45]. Longer Aβ (1–42) peptide incubations in rat models showed more toxicity in neurons [46]. Aβ peptides induced mitochondrial reductase activity, as observed in the present study, are likely to reflect a mitochondrial response within the cells of *G. pallida* which are acting against the increased oxidative stress [47,48,49].

Mitochondrial reductase activity was assessed at two different temperature conditions: ambient (18 °C) and human physiological (37 °C). All Aβ-treated groups had significantly higher mitochondrial reductase activity at 37 °C than at 18 °C. A temperature of 37 °C is considered high for *G. pallida,* and this added heat stress probably accelerated the mitochondrial responses to Aβ. A similar temperature (35 °C) has been applied to *C. elegans.* Periods of heat stress in *C. elegans* can increase life span, increase mitochondrial ROS, and enhance maintenance and repair (hormesis) [50,51,52]. This phenomenon is associated with increased expression of heat shock proteins (HSP-16 and HSP-4) which enhance the stress response in nematodes, and which may correlate with the impacts of hermetic treatments on lifespan.

Although Aβ (1–16) and Aβ (1–28) are not cytotoxic and usually do not aggregate, our results suggest that they nevertheless induce some ROS production. This is consistent with [53,54], who found that Aβ (1–16) can induce oxidative stress and increase the formation of ROS, resulting in Aβ (1–16) aggregation. However, Aβ (1–28) has been reported to not induce oxidative stress or neurotoxicity [54]. Aβ (1–40) and Aβ (17–40) induced the lowest level of ROS production, and these findings are supported by the literature describing Aβ 40-mer isoforms as being less toxic, less aggregated, and more soluble than 42-mer isoforms. Aβ (17–42) was shown to induce mitochondrial dysfunction, protein oxidation, and lipid peroxidation like Aβ (1–42) in neuronal cell cultures [54]. Also, Aβ (17–42) plays a greater role in AD pathogenesis than other fragments (e.g., Aβ (1–40)) [55]. In the present studies it was not possible to precisely assess the role of Aβ aggregation in inducing the behavioral phenotype; however, it was controlled for by freshly preparing each Aβ peptide in solution just before the experiment. Therefore, all peptides would have begun as monomers, and any peptide aggregates would have formed during the timespan of the experiment; also, it is difficult to unpick the relative contribution of aggregation and inherent cytotoxicity to each of the observed effects.

Increased ROS production causes severe damages to mitochondrial DNA and cellular injury; moreover, ROS are linked to systemic necrosis and lethality in *C. elegans* [56]. Some studies have shown that high lipid peroxidation production is induced by Aβ aggregations in AD pathology that is usually associated with increasing ROS formation. Moreover, it is thought that ROS production and Aβ leads to neuronal damage, which results in increased sensitivity to lipid peroxidation, disruption of cellular calcium homeostasis, and impairment to mitochondrial function [57,58,59].

High rates of oxidative stress increase the levels of antioxidants such as glutathione, which is an anti-oxidative defence system common in all biological processes [55,60,61,62]. It is known that parasitic nematodes use antioxidant activity as a defence against invasive plants, and ROS is believed to be the first line of defence in plants [63,64,65,66]. In the human nervous system, glutathione levels are the most abundant endogenous antioxidant molecule. On this basis, and to confirm the validity of our previous ROS results, glutathione levels were measured in control incubations of *G. pallida* or in *G. pallida* incubated in Aβ (1–42) for 24 h. The results show that Aβ (1–42) led to significantly higher GSSG/GSH compared with the control in different numbers of worms.

In a final phase of the study, we evaluated whether a range of reported neuroprotective compounds could protect *G. pallida* from impairment by Aβ (1–42). Firstly, we examined if inhibition of acetylcholinesterase (AChE) was effective in achieving this. AChE is the key enzyme that hydrolyses the neurotransmitter acetylcholine, which plays a vital role in maintaining effective synaptic communication. Reduced levels of acetylcholine can affect the communication between neurons and ultimately can contribute to cognitive decline [67,68,69]. There is an association between a decline in learning, memory, and a decreased acetylcholine in cholinergic synapses. AChE inhibitors reduce the cholinergic deficits which occur in AD patients.

Galantamine is a known AChE inhibitor which is clinically approved for treatment of mild-to-moderate AD [30,70,71,72]. Galantamine is a naturally occurring plant alkaloid isolated from the bulbs and flowers of several species, including *Galanthus caucasicus*, *Galanthus woronowii, Narcissus* (daffodil), *Leucojum aestivum*, and *Lycoris radiata*. Research indicates that there is a high level of AChE in the brain cells of AD patients [73]. Therefore, we investigated whether galantamine improves impairments to chemotaxis in Aβ-treated *G. pallida*. It was demonstrated that galantamine significantly ameliorated Aβ (1–42)-induced impairment of chemosensing.

Similarly, memantine is a known attenuator of glutamatergic (N-methyl-D-aspartate (NMDA), serotonergic (5-HT3) and cholinergic (nicotinic acetylcholine) receptors [74]. Memantine is used to treat moderate-to-severe AD, especially for people who are intolerant to or have a contraindication for AChE inhibitors. It is known to improve and protect cholinergic cells from degeneration [75]. Herein, we clearly demonstrated that memantine significantly ameliorated Aβ (1–42)-induced impairment of chemosensing. These findings agree with a previous study which employed the amyloid beta-expressing transgenic *C. elegans* [76]. A concentration of 10 mM of memantine had an anti-paralytic effect on *C. elegans* [76]. Furthermore, inhibition of both AChE and NMDA protected these nematodes from the toxic effects of Aβ (1–42) [76].

Non-clinical neuroprotective agents were also examined for their ability to ameliorate Aβ (1–42)-induced impairment of chemotaxis in *G. pallida*. Neuroprotective agents were selected based on their reported protective effects in studies of transgenic *C. elegans* AD models. First, we examined caffeine, since [77] have shown improved caffeine-induced oxidative stress resistance, which itself reduces the risk of chronic aging diseases such as AD. This study also showed that caffeine extends the lifespan of *C. elegans* [77]. Another study by [78] examined the protective effects of coffee extracts on a transgenic *C. elegans* AD model; it was suggested that coffee extracts significantly reduced the paralysis that was induced by Aβ (1–42) expression and subsequently protected against Aβ (1–42) toxicity. Second, we examined a Cocoa peptide known as 13L, which reduces paralysis resulting from Aβ (1–42) peptide expression in transgenic *C. elegans* [79]. Along with polyphenol compounds, bioactive peptides such as 13L are released from the protein fraction of cocoa [79], which possesses antioxidant properties. This could explain the reported link between dark chocolate consumption and better cognitive performance [80].

Taken together, we demonstrated that both caffeine and 13L significantly improved the CI responses of *G. pallida* treated with Aβ (1–42). The non-transgenic *G. pallida* model system provided similar results to the Aβ-expressing transgenic *C. elegans* model system. Furthermore, we showed that *G. pallida* represent a promising non-transgenic invertebrate to screen faster, easier, and reliably, for novel therapeutic compounds which protect against Aβ-induced damage.

## 5. Conclusions

In this present study, we have optimized a chemosensory assay capable of reliably measuring *G. pallida* responses. We evaluated how treatment of *G. pallida* with Aβ peptides (1–40, 1–42, 17–42, 17–40, 1–28, or 1–16) impaired the chemotaxis response to a chemoattractant and increased mitochondrial reductase activity and ROS production (leading to increased glutathione). Importantly, we have determined that, unlike *C. elegans* models, *G. pallida* assimilates Aβ (1–42), which co-localises with specific neurological structures. The ability of this unique model to distinguish between different treatment concentrations, durations, and modalities, shows that it offers remarkable sensitivity and specificity, which could be of benefit to the pharmaceutical industry. The fact that clinically approved neuroprotective agents were effective in protecting *G. pallida* from Aβ (1–42) exposure outlines its clear potential for the discovery of novel bioactive compounds with anti-AD activity.

## Figures and Tables

**Figure A1:**
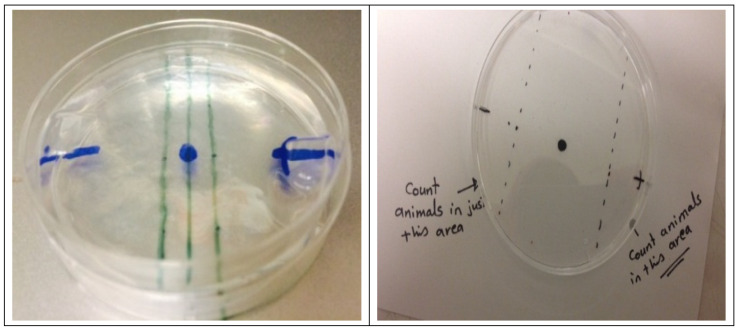
Images showing agar plates for chemotaxis assays for *G. pallida* (left; 3 cm plate) and for *C. elegans* (right; 9 cm plate).

## Data Availability

Data is contained within the article.
